# AI-Supported Digital Microscopy Diagnostics in Primary Health Care Laboratories: Protocol for a Scoping Review

**DOI:** 10.2196/58149

**Published:** 2024-11-01

**Authors:** Joar von Bahr, Vinod Diwan, Andreas Mårtensson, Nina Linder, Johan Lundin

**Affiliations:** 1 Department of Global Public Health Karolinska Institutet Stockholm Sweden; 2 Institute for Molecular Medicine Finland HiLIFE University of Helsinki Helsinki Finland; 3 Global Health and Migration Unit Department of Women’s and Children’s Health Uppsala University Uppsala Sweden

**Keywords:** AI, artificial intelligence, convolutional neural network, deep learning, diagnosis, digital diagnostics, machine learning, pathology, primary health care, whole slide images

## Abstract

**Background:**

Digital microscopy combined with artificial intelligence (AI) is increasingly being implemented in health care, predominantly in advanced laboratory settings. However, AI-supported digital microscopy could be especially advantageous in primary health care settings, since such methods could improve access to diagnostics via automation and lead to a decreased need for experts on site. To our knowledge, no scoping or systematic review had been published on the use of AI-supported digital microscopy within primary health care laboratories when this scoping review was initiated. A scoping review can guide future research by providing insights to help navigate the challenges of implementing these novel methods in primary health care laboratories.

**Objective:**

The objective of this scoping review is to map peer-reviewed studies on AI-supported digital microscopy in primary health care laboratories to generate an overview of the subject.

**Methods:**

A systematic search of the databases PubMed, Web of Science, Embase, and IEEE will be conducted. Only peer-reviewed articles in English will be considered, and no limit on publication year will be applied. The concept inclusion criteria in the scoping review include studies that have applied AI-supported digital microscopy with the aim of achieving a diagnosis on the subject level. In addition, the studies must have been performed in the context of primary health care laboratories, as defined by the criteria of not having a pathologist on site and using simple sample preparations. The study selection and data extraction will be performed by 2 independent researchers, and in the case of disagreements, a third researcher will be involved. The results will be presented in a table developed by the researchers, including information on investigated diseases, sample collection, preparation and digitization, AI model used, and results. Furthermore, the results will be described narratively to provide an overview of the studies included. The proposed methodology is in accordance with the JBI methodology for scoping reviews.

**Results:**

The scoping review was initiated in January 2023, and a protocol was published in the Open Science Framework in January 2024. The protocol was completed in March 2024, and the systematic search will be performed after the protocol has been peer reviewed. The scoping review is expected to be finalized by the end of 2024.

**Conclusions:**

A systematic review of studies on AI-supported digital microscopy in primary health care laboratories is anticipated to identify the diseases where these novel methods could be advantageous, along with the shared challenges encountered and approaches taken to address them.

**International Registered Report Identifier (IRRID):**

PRR1-10.2196/58149

## Introduction

### Background

Artificial intelligence (AI) in the form of machine learning has successfully been applied to image-based diagnostics within several medical fields [1]. Deep learning is a machine learning method that utilizes artificial neural networks that mimic the neurons of the brain and enables computers to learn and represent complex patterns in data. Convolutional neural networks (CNNs) and vision transformers are currently the most commonly used artificial neural networks for image classification and interpretation [2]. A strength of CNNs is their ability to extract features from images, such as edges or textures, which they can then use to identify complex patterns like nuclei, parasite eggs, or other structures [3]. In contrast, vision transformers split images into patches and can identify dependencies and relationships between these patches [2]. Combinations of CNNs, transformers, and other AI models have been used in attempts to leverage the strengths of different architectures [3].

Implementing AI-based diagnostics into the workflow has the potential to automate processes, increase productivity in laboratories, and improve diagnostic accuracy [4]. Multiple AI-based diagnostic systems have been approved by the US Food and Drug Administration (FDA) or corresponding authorities in Europe (notified bodies for CE marking), for example, for cervical cancer screening and prostate cancer diagnostics [4-6]. Most diagnostic systems in use utilize high-end digital imaging instruments, so called whole-slide scanners, and require access to advanced laboratory infrastructure and may, therefore, not be optimal for use in primary health care (PHC) laboratories [4,5]. However, the development of cheaper, portable digital microscope scanners has enabled research on the use of AI-supported diagnostic systems suitable for PHC laboratories [7,8].

The World Health Organization (WHO) has emphasized the importance of providing diagnostics near the patient in PHC settings, to enhance the accuracy and timeliness of diagnoses, improve clinical decision-making, and reduce the risk of diagnostic errors [9,10]. A PHC laboratory, also known as a tier 1 laboratory, can be defined as a laboratory primarily serving outpatients by providing point-of-care tests, performing slide microscopy for simple preparations, and preparing fine needle aspirations and other simple tissue specimens that are later dispatched to a tier 2 laboratory for analysis. The tier 1 laboratories work with a small budget compared to more advanced laboratories and are generally managed by a laboratory technician, supervised by a pathologist from a distance [10].

The implementation of AI-supported diagnostic systems could be particularly advantageous at PHC laboratories. To begin with, since PHC laboratories do not have access to expertise in the form of a pathologist, the application of AI could enable further analyses being performed on site, consequently increasing the availability of diagnostics [10,11]. In addition, a systematic review showed the implementation of AI-supported diagnostics for microscopy increased the effectivity of laboratory personnel [4]. Henceforth, implementation of AI could potentially lower the costs of diagnostics. Furthermore, moving diagnostics from advanced laboratories to PHC laboratories has the potential to enable faster diagnostics.

Studies have investigated the use of AI-supported microscopy in PHC settings in diagnostics for different diseases, such as oral and cervical cancers [7,12]. Furthermore, studies have targeted various parasitic infections, for example, schistosomiasis and infections caused by soil-transmitted helminths [13,14]. Although the targeted diseases differed in these studies, it appears that the researchers faced similar challenges because of the commonalities in the methodologies applied. Challenges observed when comparing these studies include the following: first, the sample preparation method needs to be simple enough to be easily performed in PHC laboratories, while maintaining sufficient sample quality, to enable AI-based analysis of the digitized sample. For example, variance in sample thickness and preparation artifacts can result in poor-quality, whole-slide images, which makes analysis with AI models challenging [8,13]. Second, the sample digitization instrument needs to be cost-efficient and easy to use in a PHC setting (with potentially unstable internet and electricity access, as well as other demanding environmental conditions) [8]. Third, the AI models need to be reliable and feasible to implement in the diagnostic workflow in a way that provides robust and accurate diagnostics. This puts a demand on acquiring training data of sufficient size and variance, as well as using appropriate AI models.

A preliminary search of the databases PubMed and Cochrane was performed to investigate whether any scoping or systematic review had been performed investigating the use of AI-supported digital microscopy in PHC laboratories. A few similar reviews were found. A systematic review of AI diagnostics for oral cancer [15] has some overlap with our proposed review, but because it investigates a single disease, it does not provide an overview of the development of AI-supported digital microscopy in PHC laboratories. A systematic review evaluating the application of AI to whole-slide images of tissue samples stained with hematoxylin and eosin (H&E) was also identified [16]. This article presents the current state of knowledge on AI implementation in pathology in high-end laboratories, highlighting different approaches regarding datasets, preprocessing of images, and different approaches to image analysis. However, to our knowledge, no scoping review has been performed to compile the present knowledge and evidence on AI-supported digital microscopy diagnostics in PHC settings.

A scoping review performed on this subject is of particular value, since multiple challenges exist that needs to be mapped and addressed to successfully implement a diagnostic system with AI-supported digital microscopy in PHC laboratories. Such a review can also provide knowledge regarding what approaches have been applied so far and guide future research toward a potential implementation of AI-supported digital microscopy in PHC laboratories.

This scoping review aims to systematically review published studies related to AI-supported digital microscopy in PHC laboratories. The scoping review will specifically address the following questions: (1) In which diseases has AI-based microscopy been applied for diagnostics within PHC laboratories? (2) What methods have been used in acquiring microscopy images to train and analyze AI models for diagnostics? (3) What AI models and training approaches have been applied? and (4) How has the AI-supported diagnostic system performed compared to expert microscopists with regard to diagnostic accuracy?

### Review Question

The research question was as follows: What studies have been published on implementing AI-supported digital microscopy in PHC laboratories? What methods have been used, what issues have been faced, and what results have been achieved?

## Methods

### Study Design

The proposed scoping review will be conducted in accordance with the JBI methodology for scoping reviews [17]. A PRISMA-ScR (Preferred Reporting Items for Systematic reviews and Meta-Analyses extension for Scoping Reviews) checklist can be found in Multimedia Appendix 1 [18]. A protocol has been published in the Open Science Framework (OSF) [19]. The inclusion and exclusion criteria are presented in Table 1.

**Table 1:** Table1. Inclusion and exclusion criteria for identified studies.

Study characteristic	Inclusion criteria	Exclusion criteria
Language	English	Not English
Study design	Diagnostic test accuracy studies	Not diagnostic test accuracy studies
Population	Humans	Animals
Concept	AI^a^ techniques applied as a diagnostic tool on microscopy Final slide-level diagnosis was performed and compared to that from a standard microscopist Outcome valuable for clinicians	Studies that applied AI models on images not conventionally analyzed in microscopy No final slide-level diagnosis
Context	Performed at a primary health care laboratory (tier 1 laboratory) No pathologist is needed on site Simple sample preparations, such as stool sample preparation, or simple tissue preparations, such as fine needle aspirations	Studies performed in an advanced laboratory setting

^a^AI: artificial intelligence.

### Eligibility Criteria

#### Participants

This scoping review will consider studies on human subjects. No exclusion will be performed based on age, sex, economic status, or nationality.

#### Concept

The studies to be included in this scoping review will have to fulfill 3 concept criteria. First, the studies need to have been performed on images gathered with an imaging instrument built to automatically capture microscopy sample areas large enough for diagnostic purposes. Further, the imaging instrument used must be operated in a way that does not require human expertise to determine what areas of the slide should be captured. Microscopy was defined as deploying a light source, optical lenses, and a digital camera to acquire a magnified image of a biological sample, generating an image conventionally interpreted by a microscopist.

Second, the studies need to have utilized AI when analyzing the microscopy images. AI was defined as a computer system that is trained to perform a task that typically requires human intelligence. No exclusion of studies will be done based on the architecture of the AI model or the dataset used for training. This analysis of the microscopy images can be performed on site or in a remote cloud environment.

Third, the studies must have compared the AI-supported diagnostic system to a standard diagnostic system. A diagnostic system was defined as all the steps included in the diagnostic process, from sample collection to the acquisition of results. The result needs to be sufficient to reach a diagnosis at the subject level.

#### Context

The included studies must have been performed in a PHC laboratory setting. To be defined as a PHC laboratory, also known as a tier 1 laboratory, the laboratory should fulfill 2 criteria. First, regarding staffing, the laboratory should be run by a laboratory technician, not requiring a pathologist on site. Second, the sample preparations should not exceed the capabilities of a PHC laboratory. Acceptable samples collected include stool, urine, blood, cytology smears, and fine needle aspirations of superficial tissue (eg, from suspected lumps in breasts). The sample staining procedure must be possible to perform manually without advanced laboratory equipment such as a microtome or tissue processor [10]. Different samples that fulfill these criteria include Kato-Katz thick stool smears, blood smears, centrifuged urine samples, Papanicolaou-stained cervical smears, and H&E-stained fine needle cytology smears [10]. Since the context of PHC laboratories is based on human medicine, the exclusion criteria and initial search strategy were changed to exclude veterinary medicine, which was included in the initial protocol published on OSF [19].

### Types of Sources

All types of diagnostic test accuracy studies will be included. Because diagnostic test accuracy studies can be both retrospective and prospective, studies using either approach will be included. Additionally, studies using both paired and random designs for reference standards will be included [20]. The included studies must be published in English.

### Search Strategy

The search strategy was designed to identify peer-reviewed published articles. An initial limited search of PubMed and Cochrane was undertaken to identify articles on the topic. Search blocks were created for the final search based on terms used in the identified articles. The search blocks were developed to find articles containing the 2 concepts, microscopy and AI, as well as the context specification of being in a PHC setting, with 1 block created for each. A detailed description of the search strategy is presented in Multimedia Appendix 2. The reference lists of all included articles will be reviewed for additional studies and duplicates. In addition, all the articles citing the included articles will be reviewed. The databases to be searched are PubMed, Web of Science, Embase, and IEEE.

### Study/Source of Evidence Selection

**Figure 1:**
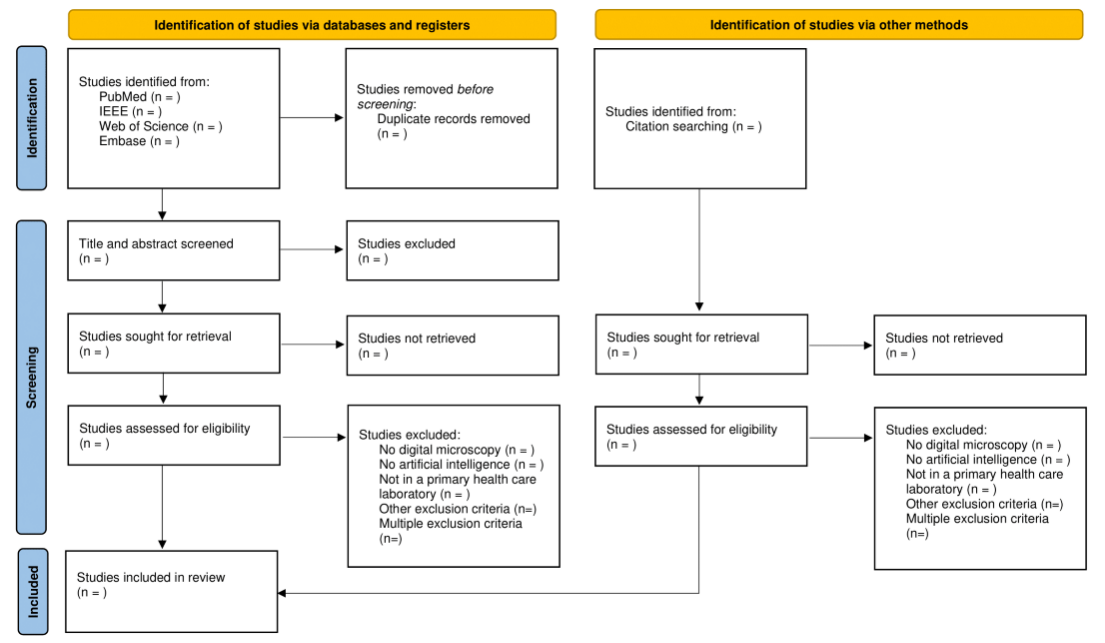
PRISMA (Preferred Reporting Items for Systematic Reviews and Meta-Analyses) flowchart for study inclusion.

Following the search, all identified articles will be compiled in a reference management software system (Zotero, version 6.0.20; January 13, 2023; Corporation for Digital Scholarship), and duplicates will be removed. Following a pilot test, titles and abstracts will be screened by 2 independent reviewers for assessment against the inclusion criteria of the review. After the exclusion based on abstracts, the full text of the remaining studies will be assessed in detail against the inclusion criteria by 2 independent reviewers. The reasons for exclusion during the full-text screening will be recorded and reported. Any disagreements that arise between the reviewers at each stage of the selection process will be resolved through discussion between the reviewers and an additional researcher. The results of the search and the study inclusion process will be reported in full in the final scoping review and presented in a PRISMA (Preferred Reporting Items for Systematic Reviews and Meta-Analyses) flow diagram (Figure 1) [21]*.*

### Data Charting and Synthesis

Data will be extracted from studies included in the scoping review by 2 independent reviewers using a data extraction tool developed by the reviewers, which can be found in Multimedia Appendix 3. Any disagreements that arise will be solved through discussion between the reviewers and an additional researcher. If any uncertainties in the data arise, the corresponding author of the manuscript will be contacted. The findings will be presented narratively and in a table format based on the extraction tool developed beforehand by the researchers. The table will have eight columns: (1) the study name, year of publication, and authors; (2) what disease the study investigated; (3) dataset preparation; (4) training procedure and dataset; (5) AI model architecture; (6) QUADAS-2 (Quality Assessment of Diagnostic Accuracy Studies-2) risk of bias; (7) validation set, results, and reference standard; and (8) additional comments. The column of dataset preparation will contain sample collection and preparation as well as digitization procedure. The narrative presentation of the results will aim to provide an overview of the results.

### Critical Appraisal of Results

To investigate the bias of the included studies, the QUADAS-2 tool will be applied. This tool was developed to assess the risk of bias for primary diagnostic accuracy studies in 4 areas: patient selection, index test, reference standard, and flow and timing [22]. The results will be presented in a table in the *Results* section, and the specific form for each article can be found in Multimedia Appendix 4.

## Results

The scoping review was initiated in January 2023, and a protocol was published in the OSF in January 2024. The protocol was completed in March 2024, and the systematic search will be conducted after the protocol has been peer reviewed. The scoping review is expected to be finalized by the end of 2024. The results of the scoping review will be disseminated through publication in a peer-reviewed journal.

## Discussion

This scoping review is anticipated to provide an overview of AI-supported digital microscopy in PHC laboratories and present what issues have been faced and measures taken to address them. In turn, the results will guide researchers who are investigating implementations of AI-supported digital microscopy by providing a disease-agnostic overview. A disease-agnostic overview of AI in microscopy has previously been conducted for H&E-stained samples in more advanced laboratories [16]. However, most scoping and systematics reviews investigating AI-supported digital microscopy in diseases that can be diagnosed outside of high-end laboratories have focused on single diseases [15,23]. Therefore, performing a study focusing on AI-supported digital microscopy for various diseases in PHC laboratories might provide novel information.

Our proposed methodology for the scoping review has several strengths. First, only studies that contain complete diagnostic systems will be considered, thus excluding studies investigating single objects (eg, cancer cells or parasite eggs) or studies containing samples gathered in an artificial setting. By doing this, the results presented in this scoping review will be more applicable to real-world settings. Second, by collating the knowledge gathered from studies performed on a multitude of diseases, this scoping review will provide a comprehensive overview of the published literature on implementing AI-supported digital microscopy in PHC laboratories.

This scoping review protocol has some limitations, for example, regarding the definition of PHC laboratories, since laboratory capabilities can vary significantly within PHC settings. To minimize bias, a definition was chosen based on the description of PHC laboratories by Fleming et al [10], excluding studies in laboratories with a pathologist on site and advanced sample preparation. By opting for a broad definition of PHC laboratories, it is anticipated that more studies will be included. This was deemed advantageous as the purpose of the scoping review is to provide an overview of the entire field; however, the settings in which the studies have been performed will probably vary to a greater extent, making comparison between studies increasingly complicated.

It is relevant to map these studies because large-scale implementation of AI-supported digital microscopy in PHC laboratories has not been achieved; however, the possibility of constructing such a system has increased with technical advancements [8,11]. Multiple studies have investigated different ways to implement AI-supported digital microscopy in PHC laboratories [7,12]. A systematic review of studies investigating AI-supported digital microscopy in PHC laboratories could highlight diseases where these novel methods could improve diagnostics, showcase the shared challenges, and present approaches taken to address them.

## Appendix

Multimedia Appendix 1 PRISMA-ScR (Preferred Reporting Items for Systematic reviews and Meta-Analyses extension for Scoping Reviews) checklist.

Multimedia Appendix 2 Search strategy.

Multimedia Appendix 3 Results table.

Multimedia Appendix 4 QUADAS-2 (Quality Assessment of Diagnostic Accuracy Studies-2) tool.

## Abbreviations

AI: artificial intelligence
CNN: convolutional neural network
FDA: Food and Drug Administration
H&E: hematoxylin and eosin
OSF: Open Science Framework
PHC: primary health care
PRISMA: Preferred Reporting Items for Systematic Reviews and Meta-Analyses
PRISMA-ScR: Preferred Reporting Items for Systematic Reviews and Meta-Analyses Extension for Scoping Reviews
QUADAS-2: Quality Assessment of Diagnostic Accuracy Studies-2
WHO: World Health Organization

## Data Availability

All data generated within this scoping review will be available when the finalized study is published, such as the QUADAS-2 (Quality Assessment of Diagnostic Accuracy Studies-2) forms and search details.
