# Supplementary material for: AI-Supported Digital Microscopy Diagnostics in Primary Health Care Laboratories: Protocol for a Scoping Review
Source: JMIR Res Protoc. 2024 Nov 1;13:e58149. doi: 10.2196/58149 (PMC11568397; doi:10.2196/58149)
Supplement: Multimedia Appendix 2 [file resprot_v13i1e58149_app2.docx]

**PubMed:** (((artificial intelligence[Title/Abstract] OR machine learning[Title/Abstract] OR deep learning[Title/Abstract] OR neural network[Title/Abstract] OR artificial neural network[Title/Abstract] OR convolutional neural network[Title/Abstract] OR generative adversarial network[Title/Abstract] OR transfer learning[Title/Abstract] OR convolution neural network[Title/Abstract] OR CNN[Title/Abstract] OR AI[Title/Abstract]) OR (ai artificial intelligence[MeSH Terms])) AND ((microscop*[Title/Abstract] OR whole slide image[Title/Abstract] OR Cytology[Title/Abstract] OR Histology[Title/Abstract] OR digital pathology[Title/Abstract]) OR (microscopy[MeSH Terms]))) AND (((point of care[Title/Abstract] OR low-cost[Title/Abstract] OR cost effective[Title/Abstract] OR resource-constrained[Title/Abstract] OR low-resource[Title/Abstract] OR primary care[Title/Abstract] OR Field based[Title/Abstract] OR Affordable[Title/Abstract] OR on-site[Title/Abstract] OR easy-to-use[Title/Abstract]) OR (point of care system[MeSH Terms])) OR (care, primary health[MeSH Terms]))

**Web of Science:** TS=(“artificial intelligence” OR “machine learning” OR “deep learning” OR “neural network” OR “artificial neural network” OR “convolutional neural network” OR “generative adversarial network” OR “transfer learning” OR “convolution neural network” OR “CNN” OR “AI”) AND TS=(microscop* OR “whole slide image” OR Cytology OR Histology OR digital pathology) AND TS=(“point of care” OR “low-cost” OR “cost effective” OR “resource-constrained” OR “low resource” OR “primary care” OR “Field based” OR Affordable OR “On-site” OR “easy-to-use”)

**IEEE:** ( ((("All Metadata": artificial intelligence OR machine learning OR deep learning OR neural network OR artificial neural network OR convolutional neural network OR generative adversarial network OR transfer learning OR convolution neural network OR CNN OR AI) OR ("Index Terms": artificial intelligence)) AND (("All Metadata": microscop* OR whole slide image OR Cytology OR Histology OR digital pathology) OR ("Index Terms": microscopy)) AND (("All Metadata": point of care OR low-cost OR cost effective OR resource-constrained settings OR primary care OR Field based OR affordable OR On-site OR easy-to-use) OR ("Index Terms": Point of care) OR ("Index Terms": Primary health care))))

**Embase:** ('artificial intelligence':ab,ti OR 'machine learning':ab,ti OR 'deep learning':ab,ti OR 'neural network':ab,ti OR 'artificial neural network':ab,ti OR 'convolutional neural network':ab,ti OR 'generative adversarial network':ab,ti OR 'transfer learning':ab,ti OR 'convolution neural network':ab,ti OR cnn:ab,ti OR ai:ab,ti OR 'artificial intelligence'/exp) AND ('microscopy'/exp OR microscop*:ab,ti OR 'whole slide image':ab,ti OR cytology:ab,ti OR histology:ab,ti OR 'digital pathology':ab,ti) AND ('point of care':ab,ti OR 'low cost':ab,ti OR 'cost effective':ab,ti OR 'resource constrained':ab,ti OR 'primary care':ab,ti OR 'field based':ab,ti OR affordable:ab,ti OR 'on-site':ab,ti OR 'easy-to-use':ab,ti OR 'point of care testing'/exp OR 'primary medical care'/exp)
