# Supplementary material for: AI-Supported Digital Microscopy Diagnostics in Primary Health Care Laboratories: Protocol for a Scoping Review
Source: JMIR Res Protoc. 2024 Nov 1;13:e58149. doi: 10.2196/58149 (PMC11568397; doi:10.2196/58149)
Supplement: Multimedia Appendix 3 [file resprot_v13i1e58149_app3.docx]

| Title, author, year | Target disease | Dataset preparation | Training procedure and dataset | AI model architecture | Quadas-2 risk of bias | Validation-set, Results and reference standard | Additional comments |
| --- | --- | --- | --- | --- | --- | --- | --- |
|  |  | How the samples were collected, prepared, and scanned | How the model was trained, the number of samples, training parameters | The structure of the AI |  | Number and origin of samples, what it was compared to and results |  |
